# Journey less travelled: Female nursing students’ experiences in providing intimate care in two nursing education institutions in Gauteng province, South Africa

**DOI:** 10.4102/hsag.v27i0.1778

**Published:** 2022-02-25

**Authors:** Simangele Shakwane

**Affiliations:** 1 Department of Health Studies, College of Human Sciences, University of South Africa, Pretoria, South Africa

**Keywords:** female nursing students, intimate care, nursing care, nursing education, nursing profession, patients, touch

## Abstract

**Background:**

Intimate care is not facilitated in South African nursing education and training. Nursing students encounter it for the first time in clinical practice, where they see and touch the naked bodies of patients. The societal segregation of gender roles has led to the feminisation of the nursing profession, suggesting that women are more caring and maternal and that intimate care implementation comes more easily to them than to their male counterparts.

**Aim:**

This study explored female nursing students’ experiences of intimate care for diverse patients.

**Setting:**

The study was conducted in two nursing education institutions in Gauteng province, South Africa.

**Methods:**

Descriptive phenomenology was used to describe the lived experiences of participants. Seventeen female nursing students were purposively sampled. Data were collected using semi-structured interviews and analysed using Moustakas’ (1994) eight steps.

**Results:**

Four themes emerged: intimate care comprehension, preparedness for providing intimate care, reactions in providing intimate care and coping mechanisms when providing intimate care to diverse patients.

**Conclusion:**

Intimate care forms a basis on which nursing students prioritise the physical needs of patients by providing care that exposes their bodies and requires touch. The students were taught to provide care with respect, maintaining patient autonomy and nursing professionalism. Unfortunately, age and gender barriers create feelings of discomfort and embarrassment. More needs to be done to support and empower nursing students in providing intimate care to diverse patients competently, confidently and comfortably.

**Contribution:**

Understanding the experiences of participants in providing intimate care to diverse patients will assist nurse educators in intimate care facilitation and support. The female nursing students will be empowered and trained to execute intimate care in a manner that is culturally, religiously and ethically acceptable.

## Introduction

Care in nursing involves assisting patients with daily health-related needs such as hygiene, elimination and nutrition, leading to intimate encounters (Mainey et al. 2018). The nursing care routines are task-oriented, aimed at promoting physical and psychological comfort where touch is essential and unavoidable (Pedrazza et al. 2018). Nurses touch patients when performing care procedures such as assistance with intimate bodily functions such as bathing and elimination (toileting), communicating care and ensuring comfort (Kelly et al. 2018; O’Lynn, Cooper & Blackwell 2017). Physical touch requires sensitivity, responsiveness and involvement from a nurse as it implies physical, cognitive and emotional proximity to the patient (Pedrazza et al. 2018). Intimate care occurs in any clinical setting where a patient needs assistance with personal care. Nursing students have to touch a patient’s body, and a patient allows a stranger to see and touch his or her fragile body. Provision of intimate care may produce feelings of discomfort, anxiety, or fear and might be misinterpreted as having sexual intent (O’Lynn et al. 2017). Thus, nurses are constantly required to negotiate boundaries and spaces (Kelly et al. 2018) during patient care to avoid discomfort for both parties.

Sexualisation of touch is different for male and female nurses; to date, female nurses’ touch is seen as natural and maternal (Kelly et al. 2018). This confirms the image of nurses as feminine, subordinate and altruistic providers of love and care (Reid-Searl et al. 2018). Caring behaviour stems from having special affection or concern for the recipient (Liu et al. 2019). This has led to the belief that female nurses are more caring and therefore, they do not require support in providing caring skills. Most of the literature focuses on male nurses’ experiences of intimate care and touch. Kelly et al. (2018) indicate that men and women as initiators and recipients of care may misinterpret touch, overshadowing the potential benefits of providing basic care and comfort.

Nursing students enter the nursing profession with their socialisation, values and beliefs about touch and intimacy; such practices are generally private and happen within the safe space of a family (Mainey et al. 2018). South African nursing students are from diverse communities, so nurse educators are challenged to facilitate methods of intimate care that are culturally and religiously acceptable (Shakwane & Mokoboto-Zwane 2020a). Providing intimate care can be stressful for nursing students; touching patients’ private body parts may evoke feelings of distress and apprehension (Mainey et al. 2018). Little emphasis is placed on how nurses should provide touch with respect and dignity (O’Lynn et al. 2017). Shakwane and Mokoboto-Zwane (2020b) attest that in South African nursing education institutions (NEIs), the socio-cultural aspects of intimate care are not emphasised during the facilitation of intimate care procedures. Yet, nursing students are expected to provide it to diverse patients.

The study explored the experiences of female nursing students in providing intimate care to diverse patients. The objectives were to:

- describe female nursing students’ perceptions of intimate care
- explore and describe nursing students’ experiences when providing intimate care to diverse patients
- identify the coping mechanism that female nursing students use when providing intimate care to diverse patients.

## Study design

A theory-generating study was conducted. This article presents the findings of female nursing students’ experiences when providing intimate care to diverse patients. A qualitative phenomenological design was used to explore these experiences. Phenomenology is a theoretical perspective that attempts to generate knowledge about how individuals experience things. It aims to examine people’s experiences of a phenomenon (Liamputtong 2013). Descriptive phenomenology was used to carefully describe the ordinary conscious experience of everyday life of people (Polit & Beck 2017). It assists in exploring human experiences through descriptions of the phenomenon provided by the individuals involved and interpretation of the experiences on what meaning the experiences hold for them (Brink, Van der Walt & Van Rensburg 2018).

The researcher used the four principles of descriptive phenomenology: bracketing, intuiting, analysing, and describing. *Bracketing* is a process of identifying and holding in abeyance preconceived beliefs and opinions (Polit & Beck 2017). During bracketing, the researcher, as a nurse educator, kept a reflective journal to write her ideas about intimate care and a literature review was conducted after data analysis to avoid bias. *Intuiting* occurs when the researcher remains open to the meanings attributed to the phenomenon by those who experience it (Greening 2019). In *analysing,* the researcher contrast and compare the final data to determine the patterns and themes (Brink et al. 2018). Data were analysed using Moustakas’ (1994) method, which requires working with the detailed data analysis results to discover essences and universal themes. Finally, *describing* is the phase where the researcher comprehends and defines the phenomenon (Greening 2019; Polit & Beck 2017). This was carried out in the discussion and critical description of the findings.

### Setting

The study was conducted in two NEIs accredited by the South African Nursing Council to provide education and training for the comprehensive nursing programme R.425 in Gauteng province. A government nursing college and a university were sampled. Both NEIs offered the R.425 nursing education programme. Intimate care procedures related to hygiene and elimination are simulated during the Fundamental Nursing Sciences (first year) and General Nursing Sciences (second and third year).

### Study population and sampling strategy

The sampling population were female nursing students registered for a comprehensive nursing education programme leading to registration as a registered nurse (R.425) in the selected NEIs in Gauteng province. Non-probability purposive sampling was undertaken to deliberately choose specific individuals who could provide crucial information about the phenomenon under study (Liamputtong 2013). Female nursing students who were registered for the R.425 nursing education and training programme and who had been allocated in general wards (medical and surgical) for 6 months or more in accredited clinical placements were selected. The female nursing students were selected to obtain information-rich data that provided an in-depth understanding of their experiences when providing intimate care to diverse patients. The sample consisted of 17 female nursing students in their second and third academic years in R.425 programme. Ten nursing students were in the second year and seven in their third academic year. The first-year female nursing students were excluded as they did not have 6 months experience in clinical placement. The fourth-year female nursing students were busy with clinical exams during the data collection period and thus they were also excluded from the study.

### Data collection

The data were collected using individual semi-structured interviews that were captured with a digital voice recorder. Non-verbal communication was recorded in the field notes. The interviews were conducted in a designated classroom in the NEIs. The duration of the interviews was 30–45 min. The interview guide was pretested by conducting two semi-structured interviews (one from each NEI). These interviews were analysed, and the questions were refined to meet the set objectives. Data were collected over three months (April – June 2016), till saturation was reached. This means that after 15 interviews, no new information was obtained. Two more interviews were conducted to confirm the saturation.

Three semi-structured questions were asked during the interviews:

- What is your understanding of intimate patient care?
- Can you describe your experiences when providing intimate care to diverse patients?
- Can you share how you coped with intimate care challenges/conflicts when caring for diverse patients?

### Data analysis

The data were analysed manually, starting verbatim transcription of the digital audio-recorded interviews. Phenomenological analysis is characterised by the procedures of bracketing, identifying common meanings and essences, horizontalisation of data and textual and structural analysis (Padilla-Diaz 2015). The researcher used a reflective journal to record her experiences during the study to identify personal judgement or bias. Horizontalisation of data was carried out by listing each relevant quote to help provide a textual description. Relevant topics were grouped into units of meaning to create core themes. Textual descriptions were accompanied by verbatim quotes. Structural denotations were devised and followed to identify the essence of the phenomenon. A literature control was conducted to validate or refute the invariant constituents (Greening 2019). The services of an independent coder were used. The researcher and coder independently analysed the data, discussed their findings, and agreed on the presented findings.

### Ethical considerations

The study received an ethical clearance certificate from the University of South Africa Health Studies Higher Degrees Committee (certificate number HSHDC/496/2015) and approval to conduct the study from the Gauteng Department of Health and the two selected NEIs. The participants were informed about the purpose and benefits of the study and that participation was voluntary. They were also made aware that they could withdraw from the study at any time. All participants signed informed consent before their semi-structured interviews.

## Measures of trustworthiness

Rigour is how a researcher demonstrates integrity and competence, a way of demonstrating the legitimacy of the research process (Liamputtong 2013). The principles of trustworthiness of Lincoln and Guba (1985) were used to enhance the quality of the study. *Credibility and authenticity* were maintained by purposively selecting the participants for their knowledge and unique experiences of intimate care. The service of an independent coder was used; the researcher and the independent coder discussed their independent analyses and agreed on the presented themes. The multiple realities described by the participants were presented using direct quotes, allowing the reader to recognise the descriptions and interpretations of the findings. *Transferability* was maintained by describing the research processes used in the study, which are sampling factors, setting, sample size, data collection and analysis. This distribution contributes to the credibility of the results, which determines their transferability to different contexts. For *dependability,* the research method was reported in detail to indicate that proper research practices were followed and that in future, the researcher could repeat the study (Polit & Beck 2017).

## Results

### Participants’ characteristics

Seventeen female nursing students between 20 and 39 years of age participated. All participants were registered for the comprehensive nursing education programme R.425. Ten participants were in the second academic year and seven in the third year. The second-year nursing students had day duty clinical experience in general nursing care (medical and surgical), whereas the third-year nursing students had both day and night clinical experiences. Two participants were already trained as Enrolled Nurses and joined the R.425 nursing education programme on the basis of Recognition of Prior Learning. Table 1 provides the summary of the participants’ characteristics.

**TABLE 1:** Participants’ description.

Participant’s code^1^	NEI code^2^	Age	Academic year	Clinical experience
FP 1	NEI 1	24	2	M & S^3^ nursing day duty
FP2	NEI 1	23	2	M & S nursing day duty
FP3	NEI 1	20	2	M & S nursing day duty
FP4	NEI 1	26	2	M & S nursing day duty
FP5	NEI 1	39	2	RPL^4^ – enrolled nurse
FP6	NEI 2	20	2	M & S nursing day duty
FP7	NEI 2	23	2	M & S nursing day duty
FP8	NEI 2	24	2	M & S nursing day duty
FP9	NEI 2	23	2	M & S nursing day duty
FP10	NEI 1	28	3	M & S nursing day and night duty
FP11	NEI 1	27	3	M & S nursing day and night duty
FP12	NEI 1	30	3	M & S nursing day and night duty
FP13	NEI 2	24	3	M & S nursing day and night duty
FP14	NEI 2	24	3	M & S nursing day and night duty
FP15	NEI 2	25	3	M & S nursing day and night duty
FP16	NEI 2	23	3	M & S nursing day and night duty
FP17	NEI 1	29	2	RPL – enrolled nurse

NEI, nursing education institutions.

1 FP – female participant.

2 NEI code – NEI 1(government nursing college), NEI 2 (university).

3 M & S – Medical & Surgical nursing.

4 RPL – Recognition of Prior learning.

### Interview results

Four themes were developed from the analyses, namely, (1) intimate care comprehension, (2) preparedness for providing intimate care, (3) reactions to providing intimate care, and (4) intimate care coping mechanisms. The sub-themes are discussed under each theme in the following subsections.

#### Theme 1: Intimate care comprehension

There were three sub-themes in which the theme of intimate care comprehension was derived. The participants’ overarching intimate care understanding was based on an individual’s ability to take care of his/her own body.

**Sub-theme 1: Care provided when a person can no longer take care of himself or herself**: Care of the sick starts in a safe environment where family members assist their loved ones with their basic needs. Once the illness is beyond their ability to care, care outside the family is sought. The participants viewed the provision of intimate care as follows:

‘Intimate care is the care at the lowest level of a person […], you are close to them and do the basic care for them which they can’t do it themselves, like brushing their teeth, bringing bedpan and urinary to them.’ (FP4, 26, NEI1)

‘Lowest level is where a person who cannot perform their basic needs, stuff that is important like hygiene, brushing teeth and not doing basics that they are expected to do.’ (FP5, 39, NEI1)

‘I think intimate care is giving care to patients who cannot do it for themselves […].’ (FP6, 20, NEI2)

**Sub-theme 2: Nursing care routines to meet the basic physical needs of a patient:** Most participants considered basic care needs such as elimination and hygiene to be intimate:

‘[*T*]ouching, giving bedpan, changing a urinary catheter, changing pampers […].’ (FP5, 39, NEI1)

‘[*B*]athing the patient, changing nappy and changing the patients’ clothes.’ (FP7, 23, NEI2)

‘[*O*]ral care and changing pampers and position, some patients when changing a position come close to you […].’ (FP6, 20, NEI1)

**Sub-theme 3: Care that involves touching and exposing patients’ private body parts:** When providing basic nursing care, the nursing students expose and touch the patients’ body parts. The majority of the participants experienced that when providing basic nursing care, touching and seeing the patient’s body is unavoidable:

‘When providing intimate care, you end up exposing the body of the patient, exposing their private part while giving care to him or her.’ (FP6, 20, NEI1)

‘I think intimate care has to do with exposing a patient, sometimes it can be basic needs like dressing the private parts of the patient, and that’s when intimacy comes, […] is where intimate care comes in.’ (FP3, 20, NEI1)

‘When you take care of a patient privately, touching them, seeing their naked bodies […].’ (FP5, 39, NEI1)

When patients receive intimate care, they are vulnerable and dependent on the care provided by a nurse.

‘… when you are bathing a patient, it’s you and the patient, and a patient is exposed and vulnerable and is under your care, and you have to protect them.’ (FP11, 27, NEI1)

Each patient is unique in the body parts that he or she considers sensitive. When providing intimate care, nurses have to listen to a patient’s needs:

‘[*I*]s based on what a patient regards as sensitive to them, the patient is not comfortable when you are holding a certain part of their bodies ….’ (FP15, 25, NEI2)

#### Theme 2: Preparedness for providing intimate care

The theme of preparedness for providing intimate care was derived from three sub-themes. These sub-themes confirm that the participants were taught basic nursing care procedural principles. These principles include respect for the patient, patient involvement and maintaining professionalism.

**Sub-theme 1:** Being respectful: The participants were taught to assess the needs of the patients, forget about themselves and act normally even if the care was difficult. This means involving patients during care and maintaining professionalism at all times. The participants said:

‘We were given an example of bathing a patient, like if a patient is a male and if there is an erection you need to act normal, you just need to treat the patient with respect. You don’t have to shout at him or what; you just need to continue with what you are doing.’ (FP6, 20, NEI2)

‘Not to expose the patient’s body unnecessarily so that they can be comfortable with us; we must ask them whether they are comfortable or not if taking off their clothes.’ (FP5, 39, NEI1)

**Sub-theme 2:** Proving information to a patient: Explaining the procedure will make the patients more comfortable during intimate care. The majority of participants expressed its importance as follows:

‘[*T*]o introduce the procedure that you are going do, make the patient understand and explain the benefits of the procedure that you are going to do, [*you must*] make them comfortable.’ (FP13, 24, NEI2)

‘[*I*]nforming them [*patients*] about the procedure eases them up and telling them [*about the*] instruments you are going to use. Telling them that this is what’s going to happen, preparing them psychologically [*assists*] them to relax.’ (FP16, 23, NEI2)

**Sub-theme 3: Maintaining professionalism:** The participants alluded to their expected behaviour during the provision of intimate care. They expressed the ability to control intimate care reactions in the following excerpts:

‘[*W*]hen performing intimate care we must act professional, don’t give a patient wrong ideas, be clear of what you are doing, […] be careful on how you touch the patient. The patient must not get the wrong idea of what you are doing, act professional.’ (FP3, 20, NWI1)

‘[*A*]cting professionally, having boundaries, you don’t give more than you have to give, you don’t give less than what you have to give. Be careful on how you touch a patient, how you communicate with the patient, be in control during patient care.’ (FP12, 30, NE1)

#### Theme 3: Reactions to providing intimate care

Two sub-themes provided the unique reactions and experiences of the participants when they were providing intimate care to diverse patients.

**Sub-theme 1:** Age barrier in caring for female patients: Some female patients refused intimate care from the participants because their young age made the patients feel uncomfortable:

‘Some patients are not comfortable because of the age, I mean, because you are young and someone [*patients*] who are older and you have to change her or bath her, so they refuse, and it becomes very difficult.’ (FP5, 39, NEI1)

‘[*A*] 65-year-old female patient had a problem being bathed by a young student nurse, she preferred an older nurse. I had to be the one to provide such care. I couldn’t render care to the patient.’ (FP13, 24, NEI2)

‘They take offence; they just feel uncomfortable as you are young, and it’s like you are taking their privacy away from them. They feel disrespected because you are a young girl. They are not willing to open up to you, and they retaliate; they become angry, starting to shout for no reason and refuse care.’ (FP4, 26, NEI1)

‘[*T*]hey feel like you are a small lady, you are a girl, they can’t be held by the student, small like you are the same age as my kid, it was harsh.’ (FP12, 30, NEI1)

**Sub-theme 2:** Discomfort and embarrassment when providing intimate care to male patients: The majority of participants had negative experiences, such as male patients taking advantage of them. Crossing the socially set gender boundaries challenges female nursing students’ ability to provide socially accepted care. This becomes problematic for them, as they must put the patient first and their values second. The participants are from diverse backgrounds, yet they share similar values, such as not looking at or touching the naked body of a man. These sentiments were expressed as follows:

‘I was told that I should not see a man naked, coming to work with a male who is naked it becomes uncomfortable because it’s something you are not used to, so it’s also affecting your relationship with a patient, based on how you were raised ….’ (FP4, 26, NEI1)

‘At home, I was taught not to see a male person’s private part, so when it came to bathing a male patient, it was a challenge, and I was scared. I cannot even talk about it at home, because it is not allowed ….’ (FP13, 24, NEI2)

‘[*D*]ue to my belief and culture […] I was taught that there is a difference between a child and an adult, a man and a woman. There are boundaries between them. You can’t see their bodies, […], you must not touch them.’ (FP1, 24, NEI1)

The provision of intimate care requires nursing students to be in close physical contact with a patient. The majority of the participants related experiences of discomfort because of patients’ physical, psychological and visual sexual advances. These experiences are discussed below:

‘You find a patient can walk, but he will call you to come and do a bath. I am not comfortable. Their facial expression makes me uncomfortable because you wonder what he is thinking about, I become uncomfortable […] they have this big weird smile or sometimes wink, I feel so scared and uncomfortable.’ (FP4, 26, NEI1)

‘I was busy helping a patient; when I tried to the other side, the patient became so silly. He touched my breast. I did not know what to do, left him and told the sister-in-charge. She told me to act professionally, and life had to go on.’ (FP6, 20, NEI2)

‘[*W*]hen I was working on the patient, I was bathing him he wanted to touch me. I first thought he needed support. I did not take it seriously. Again he started smiling and held my hand so tight that he didn’t let it go. I got so scared and uncomfortable ….’ (FP7, 23, NEI2)

While working with male patients, the most difficult moments for the participants were caring for a patient who had an erection. The excerpts below allude to the participant’s reaction to the patient’s erection during intimate care:

‘[*D*]oing bed bath to a male patient, it is difficult for the patient to contain themselves. I asked the patient to wash his private part as his hands were working; he just looked at me and put his hand on his private part. First, I thought he didn’t want me to look at it. I gave him the towel and went out when I returned; he said he could not do it. I wanted to finish [*the morning routine*]. When I moved the sheets to wash the private parts, I discovered he had an erection. I did not know what to do. I covered it and left embarrassed. My life has never been the same.’ (FP15, 25, NEI2)

‘[*D*]uring bed bath male patient had an erection, it was awkward for both of us, the patient looked naughty, and I was scared to continue, left him and [*went*] to the in-charge, but did not help, I had to finish the task. I had to cool down, go back and continue with the bed bath.’ (FP14, 24, NEI2)

#### Theme 4: Intimate care coping mechanisms

Nursing students have to cope with negative reactions or experiences during intimate care implementation. However, for the majority of the participants, even though it was difficult to provide intimate care to some patients, understanding the patient’s need for care helped them cope:

‘[*K*]eeping in mind that the patient needs me and if I don’t help him or her who will? As much as I am uncomfortable, I tell myself that if I do not help the patient, no one will ….’ (FP4, 26, NEI1)

‘[*W*]e sometimes get tired, but we have to do it because the patient needs the care. At the end of the day, it is about helping the patients and making them comfortable, protect them [*patients*] and yourself ….’ (FP11, 27, NEI1)

There is little support received from nurse educators to cope with intimate care challenges. The majority of participants described their experiences of sharing intimate care conflicts with nurse educators as follows:

‘[*W*]e discuss it in class, we joke about it. The only advice we have been given is that call someone else […] in the ward everyone is uncomfortable, and you have to do it […].’ (FP4, 26, NEI1)

‘[*E*]xamples are given, we are told […] keep doing it, and we can get used to it, we will be fine at the end of the day.’ (FP6, 20, NEI2)

‘[*T*]hey [*nurse educators*] just teach us, you have to do this and that. At the end of the day, you are alone with the patient and uncomfortable. They just teach us the basics according to the curriculum.’ (FP11, 27, NEI1)

‘You have to deal with it alone. Our facilitators tell us that it will be much better as years pass by, and it’s part of nursing. They don’t know what to say to us; we are burdened with this care.’ (FP2, 23, NEI1)

The sensitive nature of intimate care made it difficult for some participants to freely share their experiences with the nurse educators and fellow students. Some comments were:

‘I find intimate care as something that is not important, so I don’t say much about it. We share about cancer and trauma cases, but intimate care we don’t deal much about it.’ (FP16, 23, NEI2)

‘I can’t share in class that I can’t handle this intimate care […] fearing to become a laughing-stock as it is private. You end up having to deal with it yourself […].’ (FP15, 25, NEI2)

‘If you share with your guardian lecturer, it seems as if you are weak and you don’t have the call and the abilities to be a nurse. I don’t talk about it anymore […].’ (FP13, 24, NEI2)

## Discussion

The study aimed to explore the lived experiences of female nursing students when providing intimate care to diverse patients.

### Intimate care comprehension

Intimate care was perceived to be the care provided when patients can no longer care for themselves; thus, this is a care generally occurred within the family’s safety. De Beer and Brysiewicz (2017) assert that in a South African context, family care embraces the principle of ubuntu, where a family provides care as a sign of compassion and caring. It is hard for individuals to be dependent on others for basic human necessities, which may lead to feelings of being a burden to others and psychological distress (Thompson et al. 2021).

The participants also viewed intimate care as those nursing tasks required to care for a patient’s basic physical needs. Intimate care activities are basic physical care that is necessary for daily living, which may evoke strong emotions in individuals who are recipients of such care (Thompson et al. 2021). Mainey et al. (2018) consider intimate care as fundamental nursing care routines such as hygiene, elimination, and nutrition to assist patients with their physical needs. These procedures are often considered simple, humble tasks and carry less prestige (Thompson et al. 2021).

Intimate care also involves physical and psychological closeness between a nurse and a patient. The provision of intimate care transforms private and personal activities into a social process shaped by complex beliefs, behaviours, attitudes and cultures (Thompson et al. 2021). The nurse comes into contact with a patient’s body, and the patient allows a nurse to touch his or her body (Shakwane & Mokoboto-Zwane 2020a). Touch cannot be avoided when caring for the patient’s body and often it includes exposing a patient’s sensitive body parts. O’Lynn and Krautscheid (2011) define intimate care as task-oriented touch to areas of patients’ bodies that might evoke feelings of discomfort, anxiety and fear or might be misinterpreted as having a sexual purpose.

### Preparedness for providing intimate care

During the simulation of clinical procedures, the participants were taught to show respect, provide patients with care information, and maintain professionalism. Respect requires a nurse to treat patients with regard, concern, protect their privacy, be sensitive to their diverse culture, and allow them to make choices (Umbreen & Jabeen 2019). This is further stated by Zirak, Ghafourifard and Aliafsari Mamaghani (2017) that respect assists one to develop self-worth through maintaining dignity, protecting patients’ privacy and allowing them to have their autonomy.

Providing information to the patient about what will be performed is essential. According to O’Lynn and Krautscheid (2011), patients expected nurses to communicate the nature of the procedure to be performed, seek permission and be involved in the execution of intimate care. Nursing professionalism encourages nursing students to respect the patients and create boundaries that protect the profession’s image. Professionalism conceptualises the obligations, attributes, and interactions concerning the patients and society (Fantahun et al. 2014). Shakwane and Mokoboto-Zwane (2020b) emphasise a therapeutic nurse–patient intimate relationship that is based on trust, respect and dignity. This relationship increases patient satisfaction and acceptance of care (Hosseini et al. 2019).

### Reactions to providing intimate care

Experiences of providing intimate care are guided by the patient’s age, gender and sexual orientation. When providing intimate care to female patients, the majority of participants experienced refusal of care and discomfort because of the age difference. Nursing students are novices in age, social maturity and responsibility (Crossan & Mathew 2013), and age is pivotal in accepting and rejecting intimate care (Shakwane & Mokoboto-Zwane 2020a). Some female patients refused intimate care because the participants were young. Yet, Crossan and Mathew (2013) found that nurses even had challenges while providing intimate care to patients of the same age.

Caring for male patients created discomfort and embarrassment. The participants described their values of not seeing or touching the naked body of a man. Nursing students have socio-cultural values and beliefs in which touch and intimacy are private and not spoken of, and they have to view a sick person as a patient only (Crossan & Mathew 2013). With the increased diversity in healthcare, nurses are expected to provide sensitive and appropriate care to all patients. Providing effective and culturally sensitive care requires nurses to be aware of personal and patient’s cultural and social beliefs (Shahzad et al. 2021). Nursing students should be prepared on transcultural issues related to caring for the human body.

Some patients make sexual advances during intimate care. Sexual harassment is defined as unwelcome sexual behaviour, including verbal, physical, psychological or visual forms (Chang, Tzeng & Teng 2020). Kahsay et al. (2020) attest that sexual harassment of female nurses by male patients is prevalent; about 31% is physical and 40.8% psychological (verbal and visual). The authors explain that nurses’ exposure to harassment is because of their work bringing them to physical closeness with the patients. For nursing students, sexual harassment may lead to deterioration in physical and mental health (Chang et al. 2020). Penile erection during intimate care is not well researched; most literature focuses on male patients’ reproductive health dysfunctions and oncology problems. Nursing students should be prepared for such challenges regardless of the causes to recognise the risks surrounding intimate care and be equipped for the procedure (Reid-Searl et al. 2018).

### Intimate care coping mechanism

For the participants to cope with the challenges of intimate care, they put the needs of patients first. This is congruent with the study of Crossan and Mathew (2013) that female nursing students sought to prioritise the comfort and privacy needs of the patients. Many studies have discussed strategies used by male nurses to cope with intimate care challenges, such as using female nurses as chaperones (Whiteside & Butcher 2015) and respecting patients’ gender preferences (O’Lynn & Krautscheid 2011). It is acceptable for female nurses to care for all patients irrespective of their gender (Crossan & Mathew 2013). In contrast, male nurses take overt cautious care to avoid potential accusations that might result from the misinterpretation of intimate care (O’Lynn et al. 2017). However, the participants received little support and feared disapproval if they expressed difficulties associated with intimate care. Nurse educators focus clinical simulations on hygiene and elimination psychomotor aspects, neglecting the affective intimate care skills such as touch and navigating personal body space in practice (Mainey et al. 2018). Lack of intimate care instruction leads to the care being taken for granted and nursing students not being prepared for or supported in providing such care competently, confidently and comfortably (Shakwane & Mokoboto-Zwane 2020b).

### Limitations

The study was conducted in Gauteng province in South Africa. Because of the sensitive nature of intimate care, some participants may have withheld valuable information. Future studies should be conducted using different methods and in more provinces of South Africa.

### Recommendations

Intimate care should be considered important, and female nursing students should be prepared for providing it. Further studies should be conducted to explore the influence of culture in intimate care provision and examine sexual harassment and reporting systems for nursing students in South Africa.

## Conclusion

The current study demonstrates the value of intimate care, its challenges for female nursing students and the need for continuous support. Intimate care addresses the basic physical and psychological needs of a patient. The participants experienced rejection because of their youthfulness, inexperience, and physical and psychological sexual advances when executing this care. Lack of support and fear of mentioning intimate care conflicts may lead to discontinuity in quality patient care. The findings suggest that teaching intimate care will prepare female nursing students to assess intimate care risks and handle patients’ bodies.
